# Nuclear imaging methods for the prediction of postoperative morbidity and mortality in patients undergoing localized, liver-directed treatments: a systematic review

**DOI:** 10.1186/s13550-020-00687-1

**Published:** 2020-09-04

**Authors:** Caroline Espersen, Lise Borgwardt, Peter Nørgaard Larsen, Trine Borup Andersen, Louise Stenholt, Lars Jelstrup Petersen

**Affiliations:** 1 Department of Clinical Physiology, Nuclear Medicine and PET, Rigshospitalet, Blegdamsvej 9, DK-2100 Copenhagen, Denmark; 2 Department of Gastrointestinal Surgery, Rigshospitalet, Blegdamsvej 9, DK-2100 Copenhagen, Denmark; 3 Department of Clinical Medicine, University of Aalborg, Sdr. Skovvej 15, DK-9000 Aalborg, Denmark; 4 The Medical Library, Aalborg University Hospital, Sdr. Skovvej 15, DK-9000 Aalborg, Denmark; 5 Department of Nuclear Medicine, Clinical Cancer Research Center, Aalborg University Hospital, Hobrovej 18-22, DK-9100 Aalborg, Denmark

**Keywords:** Liver nuclear imaging, Liver function, Liver resection, Liver failure, Mortality

## Abstract

**Background:**

Several nuclear imaging methods may predict postoperative liver function and outcome, but none has achieved recommendations in clinical guidelines. The purpose of this systematic review was to summarize the existing knowledge on this topic.

**Methods:**

MEDLINE and Web of Science were searched for studies investigating nuclear medicine imaging methods for the prediction of postoperative liver function in patients undergoing localized, liver-directed treatments. The postoperative endpoints were clinical outcome (morbidity and mortality) as well as measures of postoperative liver function, e.g., liver function assessed by biochemical tests or nuclear imaging.

**Results:**

A total of 1352 references were identified, of which 82 fulfilled the eligibility criteria and were included in the review. Most studies (*n* = 63) were retrospective studies. The vast majority of studies assessed [^99m^Tc]Tc-galactosyl serum albumin (GSA) (*n* = 57) and [^99m^Tc]Tc-mebrofenin (*n* = 19). Liver resection was entirely or partly major (involved at least three segments) in 78 reports. There were notable variations in the research methodology, e.g., image acquisition, imaging variables, and endpoints. Thirty-seven studies reported on postoperative mortality, of which most reported descriptive data at the patient level. Of the four reports that performed multivariate analyses, two showed significant predictive results of isotope-based preoperative tests. Fifty-two papers presented data on postoperative liver failure. Multivariate predictive analyses were performed in eighteen trials, of which fifteen showed the significant value of nuclear medicine tests.

**Conclusion:**

There is sparse evidence supporting the significant value of nuclear medicine imaging methods in predicting postoperative mortality. In contrast, a notable number of trials showed a significant prediction of liver failure in multivariate analyses. The research methodology was heterogeneous and exploratory in most trials. Documentation of nuclear medicine tests in this setting awaits the results of properly designed, prospective trials with the standardization of both the nuclear medicine test and endpoints.

## Background

Despite the advancement and improvement of surgical techniques and perioperative management over recent years [1], major liver resection still bears the risk of inducing postoperative liver failure (LF) or other major liver-related complications, especially in patients with underlying parenchymal liver disease [2, 3]. LF is a serious complication following liver resection and the major cause of postoperative mortality and morbidity [4, 5]. However, liver resection remains the best curative method for treating colorectal liver metastases [6, 7] and primary hepatobiliary cancers [7]. Insufficient remnant liver function is an important factor contributing to a poor postoperative outcome [6]. Therefore, any procedure with the purpose of removing large amounts of diseased liver tissue should include a pre-procedural risk assessment by estimating the future liver remnant (FLR) function to avoid post-procedure LF, mortality, or other liver-related complications.

Both computed tomography (CT) and biochemical liver function tests have been employed in the preoperative assessment by measuring the volume of the FLR and the global liver function, respectively [8]. CT is presently the gold standard for the preoperative evaluation of the FLR [9]. Using CT volumetry as an indirect assessment of FLR function assumes that the liver function is homogenously distributed. However, as patients often present with compromised livers with heterogeneously distributed liver function, this assumption is not always true. Therefore, estimating the function of the FLR directly may be more reliable in predicting the real postoperative remnant liver function rather than estimating the volume or global liver function [8, 10, 11].

With the use of specific nuclear imaging techniques, it is possible to evaluate the function of the FLR directly. Presently, there are no guidelines or definite, widely accepted recommendations on liver function assessment with nuclear imaging methods prior to a procedure with the purpose of removing or destroying diseased, localized liver parenchyma. Therefore, it would be of great clinical value to establish a reliable, noninvasive method with specific guidelines and cut-off values for the preoperative assessment of postoperative risk in patients undergoing liver resection.

The purpose of this systematic review was to investigate the clinical documentation of preprocedural nuclear imaging methods to predict postprocedural clinical outcomes after local intervention in the liver, including the prediction of LF and death.

## Materials and methods

### Literature search strategy

The literature search was performed by a trained research librarian (LS) using two bibliographic databases, MEDLINE (Ovid) and Web of Science Core Collection (Clarivate Analytics). The search period spanned from the start date of each database until May 27, 2020. Due to the extensive amount of work preparing this review, the original search was outdated, and a new search was performed (as of May 27, 2020) in order to ensure that no relevant studies meeting the criteria for inclusion were left out. The literature search was customized for each bibliographic database and set up to match the predefined PICOS criteria (patient, intervention, comparison, outcome, study design) (Supplementary file 1). In brief, we searched for original papers where a nuclear medicine imaging examination was performed prior to any intervention for localized liver disease, and the results of the imaging assessment were compared to a clinical outcome. Using both controlled thesaurus terms and natural language terms to include synonyms, the search terms included descriptions of the underlying liver condition, the liver intervention, and the liver nuclear imaging technique (see Supplementary file 2 for the exact search profiles). All the identified references were run through a reference managing software tool (EndNote X9, Clarivate Analytics, Philadelphia, USA) to identify duplicate studies. The unique references were entered into a systematic review management system (Covidence, Veritas Health Innovation, Melbourne, Australia), which was used for title/abstract and full-text screening of the studies. There is no public protocol registration for this systematic review. The systematic review was conducted in full accordance with the Preferred Reporting Items for Systematic Reviews and Meta-Analyses (PRISMA) guidelines [12].

### Eligibility criteria

The eligibility criteria were as follows. (1) The included patients had to undergo local treatment for focal or multifocal liver disease with an intent to eliminate the diseased liver tissue, irrespective of the underlying cause of disease. (2) The interventions included both liver interventions and preprocedural imaging interventions. The liver interventions included but were not limited to surgery, radiotherapy, cryotherapy, percutaneous ethanol injection, percutaneous microwave coagulation therapy, radiofrequency ablation, or transcatheter arterial chemoembolization. The imaging interventions involved the preprocedural assessment of liver function with a nuclear medicine imaging method. (3) There was no requirement of any comparator. (4) The outcome had to be correlated to the preprocedural nuclear imaging technique and included reporting of postoperative mortality, LF, postoperative complications, and/or postoperative liver function tests (e.g., biochemical tests or nuclear imaging techniques). (5) Any study design with a minimum of five patients per study was considered (Supplementary file 1).

### Study selection

Two investigators independently performed the review of the studies. Initially, the original studies retrieved from the literature search were reviewed for eligibility based on reading the titles and abstracts. Only if the two investigators agreed upon exclusion at this level, the paper was discarded. The full texts of all the remaining papers were subsequently read. The systematic review included studies meeting all eligibility criteria as judged by both investigators.

### Data extraction

The following data were extracted: country (affiliation of the first author), year of publication, patient enrolment (prospective/retrospective), patient selection (consecutive/nonconsecutive), number of included patients undergoing liver intervention and nuclear imaging who were followed for outcome, type of liver intervention, nuclear medicine imaging technique, and outcome reporting. The number of included patients only encompasses the patients undergoing a preprocedural nuclear imaging examination and an intervention with the purpose of removing diseased liver tissue. Patient enrolment was characterized as prospective if the word “prospective” was used, the terminology was clear, it was described as an interventional design, informed consent was obtained individually before participation in the study, and/or the study partly comprised healthy volunteers (even without providing information about informed consent or ethical approval). Patient selection was classified as consecutive if the word “consecutive” was used or it was clear that all patients meeting certain eligibility criteria in a well-defined time period were included in the study. The surgical procedure was characterized as a major or minor surgery according to the Couinaud criteria (resection ≥ 3 liver segments vs. 1–2 segments) [13].

It was recorded whether the study reported outcome data categorized as mortality, LF, other clinical morbidity, or liver function tests (yes/no option) if they correlated the preoperative nuclear medicine test to the postoperative outcome. Due to the large number of papers reporting high-level outcomes (mortality and LF), detailed data extraction was performed for these two categories only. The outcome data were extracted provided the study included data for a preprocedural nuclear imaging examination and postprocedural mortality or LF. If data on mortality and LF were reported independently, the data were extracted for each of these outcomes separately. If a study analyzed a postprocedural composite endpoint, e.g., overall complications including mortality or LF, the mortality and/or LF data were extracted if possible and reported separately if preprocedural nuclear imaging data were reported separately for those deceased and for those who developed LF. Otherwise, the outcome was shown as a composite outcome and reported in either the mortality or liver failure table or both depending on the number of events for each outcome. The outcome data were reported in the mortality table if all patients with LF died or in the LF table if the majority of these patients developed LF, of which a few died. If the definition of LF included death without other cause, mortality events due to LF were only reported in the LF outcome table. Last, if the paper reported on LF-related complications or signs of postoperative LF, it was included in the liver failure outcome table.

The following data were also extracted: (1) the definitions of mortality (e.g., all-cause mortality or in-hospital mortality) and LF; (2) the outcome event rate; (3) whether a study analyzed a predefined cut-off value of the nuclear imaging examination in a prospective setting or if the study established a post hoc cut-off value based on the observed results; (4) the diagnostic characteristics of the cut-off value as well as comparisons between the uptake values of the nuclear imaging examination in patients with or without an outcome; and eventually (5) available data regarding the predictive value of the nuclear imaging examination and outcome based on univariate or multivariate analyses in logistic or cox regression models.

### Statistics

Descriptive statistics comprised the calculation of the median and range. No analytical statistics were used.

### Ethics

In compliance with national legislation, no ethical approval or informed consent was obtained for this systematic review, as it only contains data from previously published articles and no individual data.

## Results

### Literature search and review

**Fig. 1:**
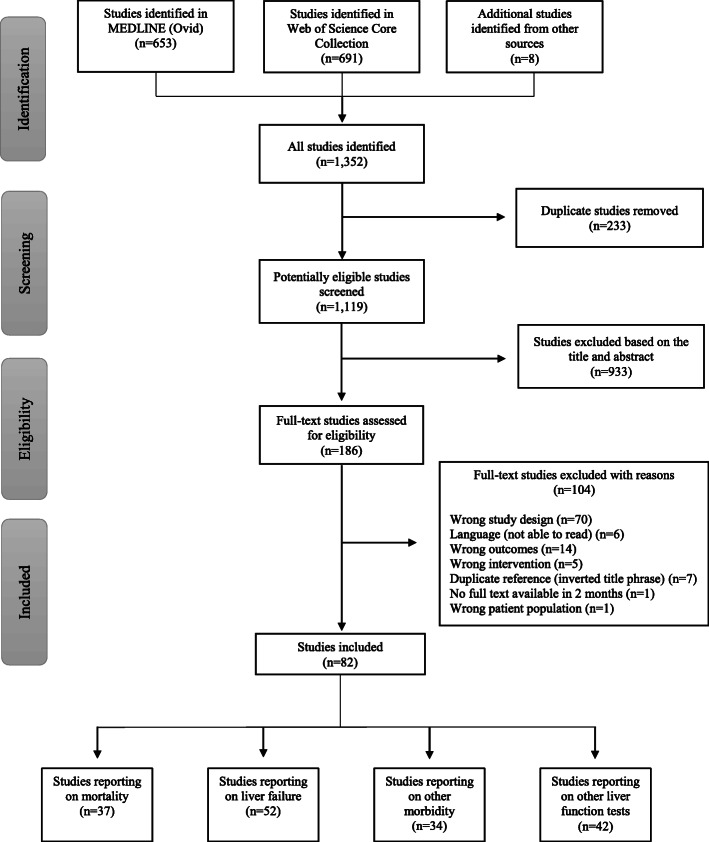
Consort flow diagram of the article selection process

A total of 1344 studies were retrieved from the literature search, and eight studies were retrieved from other sources (Fig. 1). After the removal of duplicates, 1119 studies were screened. Based on the title and abstract screening, 933 studies were excluded, leaving 186 studies for full-text screening, of which 82 eligible studies were included in the systematic review.

### Study demographics

The majority of the included studies investigated the use of [^99m^Tc]Tc-GSA (57 studies), and the majority of those trials originated from Japan (56 studies) (Table 1). Nineteen studies investigated the use of [^99m^Tc]Tc-mebrofenin. The remaining six studies employed the following tracers: [^18^F]FDG, [^99m^Tc]Tc-Sn colloid, [^99m^Tc]Tc-colloid, [^99m^Tc]Tc-PMT, L-[methyl-^11^C]-methionine, and [^198^Au]Au colloid + [^99m^Tc]Tc-Sn colloid. The vast majority of the studies were retrospective, and only nineteen papers were prospective. Forty-seven studies explicitly defined consecutive recruitment of patients. The year of publication spanned four decades from 1989 to 2020 (median 2010). The median number of included patients undergoing both a preprocedural nuclear medicine imaging examination and a procedure with the purpose of eliminating diseased liver tissue was 67 (5 to 625 patients). Some studies investigated major surgery exclusively including some studies investigating associating liver partition and portal vein ligation for staged hepatectomy (ALPPS) [21, 69, 72, 80, 82, 83]. The majority of the studies investigated both major and minor surgery. Four studies also included non-operative interventions such as radiofrequency ablation, transarterial embolization or chemoembolization, percutaneous ethanol injection, or microwave coagulation therapy [31, 52, 75, 87]. The vast majority of trials reported on postoperative LF or mortality. Sixteen papers reported solely on postprocedural morbidity and/or nonclinical outcome (liver function assessed by imaging or laboratory/biochemical tests) (Table 1), and an additional 48 studies reported on such data along with data on LF and/or mortality.

**Table 1:** Study demographics of the included papers

Author	Year	Country	Prospective study	Consecutive recruitment	Tracer	Patients (***n***)	Postoperative outcome (correlated to NMT)				Major/minor surgery or both
							Mortality	Liver failure	Morbidity	Liver function tests	
Bennink et al. [14]	2004	The Netherlands	Yes	Yes	[^99m^Tc]Tc-Meb	15	No	No	No	Yes	Both
Beppu et al. [15]	2018	Japan	Yes	No	[^99m^Tc]Tc-GSA	40	No	No	No	Yes	Major
Cieslak et al. [16]	2016	The Netherlands	No	Yes	[^99m^Tc]Tc-Meb	163	Yes	Yes	Yes	No	Major
Cieslak et al. [17]	2017	The Netherlands	No	Yes	[^99m^Tc]Tc-Meb	46	No	Yes	No	No	Major
Chiba et al. [18]	2017	Japan	No	Yes	[^99m^Tc]Tc-GSA	123	Yes	Yes	No	No	Both
Chapelle et al. [19]	2016	Belgium	Yes	Yes	[^99m^Tc]Tc-Meb	88	Yes	Yes	No	No	Both
Chapelle et al. [20]	2017	Belgium	Yes	Yes	[^99m^Tc]Tc-Meb	188^1^	Yes	Yes	No	Yes	Both
Chapelle et al. [21]	2017	Belgium	No	Yes	[^99m^Tc]Tc-Meb	140	No	Yes	No	Yes	Both
Cho et al. [22]	2017	Korea	No	Yes	[^18^F]FDG	149	No	Yes	No	No	Both
Das et al. [23]	2001	Japan	No	Yes	[^99m^Tc]Tc-GSA	100	Yes	No	Yes	No	Both
de Graaf et al. [11]	2010	The Netherlands	No	Yes	[^99m^Tc]Tc-Meb	36	No	No	No	Yes	Both
de Graaf et al. [10]	2010	The Netherlands	No	Yes	[^99m^Tc]Tc-Meb	55	No	Yes	Yes	Yes	Major
Dinant et al. [24]	2007	The Netherlands	Yes	Yes	[^99m^Tc]Tc-Meb	46	Yes	Yes	Yes	No	Both
Franken et al. [25]	2020	The Netherlands	No	Yes	[^99m^Tc]Tc-Meb	191	Yes	Yes	Yes	No	Both
Fujioka et al. [26]	1999	Japan	No	No	[^99m^Tc]Tc-GSA	15	Yes	No	Yes	No	Both
Guiu et al. [27]	2017	France	No	Yes	[^99m^Tc]Tc-Meb	9	Yes	Yes	Yes	Yes	Major
Hayashi et al. [28]	2015	Japan	No	Yes	[^99m^Tc]Tc-GSA	133	Yes	Yes	Yes	No	Major
Hino et al. [29]	1997	Japan	No	No	[^99m^Tc]Tc-Sn colloid	47	Yes	Yes	No	No	Both
Hirai et al. [30]	2003	Japan	No	Yes	[^99m^Tc]Tc-GSA	30	No	Yes	No	Yes	Major
Hwang et al. [31]	1999	Japan	Yes	Yes	[^99m^Tc]Tc-GSA	75	No	Yes	No	No	Both^2,3^
Iimuro et al. [32]	2010	Japan	No	No	[^99m^Tc]Tc-GSA	32	No	No	Yes	Yes	Both
Jansen et al. [33]	1990	The Netherlands	No	No	[^99m^Tc]Tc-colloid	11	No	No	No	Yes	Both
Kaibori et al. [34]	2008	Japan	Yes	Yes	[^99m^Tc]Tc-GSA	191	Yes	Yes	No	No	Both
Kamohara et al. [35]	2011	Japan	No	No	[^99m^Tc]Tc-GSA	180	No	Yes	Yes	No	NR^4^
Kato et al. [36]	2018	Japan	No	Yes	[^99m^Tc]Tc-GSA	100	No	Yes	No	Yes	Both
Katsuramaki et al. [37]	2003	Japan	No	Yes	[^99m^Tc]Tc-GSA	63	No	Yes	Yes	No	Both
Kawamura et al. [38]	2008	Japan	No	No	[^99m^Tc]Tc-GSA	99	No	Yes	No	Yes	Both
Kim et al. [39]	1997	Japan	No	Yes	[^99m^Tc]Tc-GSA	30	Yes	Yes	Yes	No	Both
Kokudo et al. [40]	2002	Japan	No	Yes	[^99m^Tc]Tc-GSA	111	No	Yes	Yes	No	Minor
Kwon et al. [41]	1995	Japan	No	Yes	[^99m^Tc]Tc-GSA	36	No	No	No	Yes	Both^2^
Kwon et al. [42]	1997	Japan	No	Yes	[^99m^Tc]Tc-GSA	90	Yes	No	No	No	Both^2^
Kwon et al. [43]	2001	Japan	No	Yes	[^99m^Tc]Tc-GSA	47	Yes	No	No	Yes	Both^2^
Kwon et al. [44]	2004	Japan	No	No	[^99m^Tc]Tc-GSA	32	Yes	No	No	Yes	Both
Kwon et al. [45]	2004	Japan	No	No	[^99m^Tc]Tc-GSA	152	Yes	Yes	No	No	Both^2^
Kwon et al. [46]	2006	Japan	No	Yes	[^99m^Tc]Tc-GSA	178	Yes	No	No	Yes	Both^2^
Li et al. [47]	2003	Japan	Yes	No	[^99m^Tc]Tc-GSA	44	No	No	No	Yes	Both^4^
Mao et al. [48]	2015	China	Yes	No	[^99m^Tc]Tc-GSA	69	No	Yes	Yes	Yes	Both
Mitsumori et al. [49]	1998	Japan	No	No	[^99m^Tc]Tc-GSA	20	No	Yes	No	Yes	Both
Nakamura et al. [50]	2018	Japan	No	Yes	[^99m^Tc]Tc-GSA	218	No	Yes	No	No	Both
Nakano et al. [51]	1999	Japan (and France)	No	Yes	[^99m^Tc]Tc-GSA	18	No	Yes	Yes	Yes	Major^5^
Nakano et al. [52]	1999	Japan (and France)	No	Yes	[^99m^Tc]Tc-GSA	64	No	No	Yes	Yes	Both^3^
Namieno et al. [53]	1997	Japan	Yes	No	[^99m^Tc]Tc-PMT	8	Yes	No	No	Yes	Both
Nanashima et al. [54]	2004	Japan	No	No	[^99m^Tc]Tc-GSA	140	No	Yes	Yes	No	Both^2^
Nanashima et al. [55]	2007	Japan	No	Yes	[^99m^Tc]Tc-GSA	185	No	Yes	Yes	No	Both
Nanashima et al. [56]	2010	Japan	No	Yes	[^99m^Tc]Tc-GSA	250	No	Yes	Yes	No	Both
Nanashima et al. [57]	2013	Japan	No	No	[^99m^Tc]Tc-GSA	442	No	Yes	Yes	No	Both
Nanashima et al. [58]	2013	Japan	No	No	[^99m^Tc]Tc-GSA	67	No	Yes	Yes	No	Both^2^
Nanashima et al. [59]	2020	Japan	No	No	[^99m^Tc]Tc-GSA	229	No	Yes	Yes	No	Both
Nishikawa et al. [60]	2015	Japan	No	Yes	[^99m^Tc]Tc-GSA	213	Yes	No	Yes	No	Both
Nishiyama et al. [61]	2003	Japan	No	No	[^99m^Tc]Tc-GSA	20	Yes	No	No	No	Major
Nitta et al. [62]	2019	Japan	No	Yes	[^99m^Tc]Tc-GSA	60	No	Yes	Yes	Yes	Both
Okabayashi et al. [63]	2017	Japan	Yes	Yes	[^99m^Tc]Tc-GSA	185	Yes	Yes	Yes	Yes	Both
Okabe et al. [64]	2014	Japan	Yes	Yes	[^99m^Tc]Tc-GSA	625	No	Yes	No	No	Both^2^
Olthof et al. [65]	2017	The Netherlands	No	Yes	[^99m^Tc]Tc-Meb	116	Yes	Yes	Yes	No	Major
Otsuki et al. [66]	1997	Japan	Yes	No	[^11^C]C-Met	11	Yes	No	No	No	Major
Rassam et al. [67]	2019	The Netherlands	No	Yes	[^99m^Tc]Tc-Meb	71	Yes	Yes	Yes	No	Both
Satoh et al. [68]	2003	Japan	No	No	[^99m^Tc]Tc-GSA	57	Yes	Yes	No	No	Both
Serenari et al.^6^ [69]	2018	Argentina	No	Yes	[^99m^Tc]Tc-Meb	20	Yes	Yes	No	No	Major
Serenari et al. [70]	2019	Italy	No	Yes	[^99m^Tc]Tc-Meb	27	No	Yes	No	No	Major
Shimizu et al. [71]	2010	Japan	Yes	No	[^99m^Tc]Tc-GSA	86	No	No	No	Yes	Both^2^
Sparrelid et al .[72]	2017	Sweden	Yes	No	[^99m^Tc]Tc-Meb	9	No	No	No	Yes	Major
Sugai et al. [73]	2000	Japan	No	No	[^99m^Tc]Tc-GSA	11	No	Yes	Yes	Yes	Major
Sumiyoshi et al. [74]	2016	Japan	No	No	[^99m^Tc]Tc-GSA	30	No	Yes	No	Yes	Major
Sumiyoshi et al. [75]	2018	Japan	No	Yes	[^99m^Tc]Tc-GSA	13	Yes	Yes	Yes	No	Both^3^
Takeuchi et al. [76]	1999	Japan	No	Yes	[^99m^Tc]Tc-GSA	40	Yes	No	Yes	Yes	Both
Tanabe et al. [77]	1989	Japan	No	No	[^198^Au]Au colloid + [^99m^Tc]Tc-Sn colloid	21	Yes	No	Yes	No	Both
Tanaka et al. [78]	1999	Japan	No	No	[^99m^Tc]Tc-GSA	44	No	No	No	Yes	Both
Tanaka et al. [79]^7^	2013	Japan	Yes	Yes	[^99m^Tc]Tc-GSA	247	Yes	Yes	No	No	Major
Tanaka et al. [80]	2015	Japan	No	No	[^99m^Tc]Tc-GSA	64	No	No	No	Yes	Both
Tanoue et al. [81]	2019	Japan	No	No	[^99m^Tc]Tc-GSA	247	No	Yes	Yes	Yes	Both
Truant et al. [82]	2016	France	No	No	[^99m^Tc]Tc-Meb	5	Yes	Yes	No	Yes	Major
Truant et al. [83]	2017	France	Yes	Yes	[^99m^Tc]Tc-Meb	100	Yes	Yes	No	Yes	Major
Uetake et al. [84]	1999	Japan	No	No	[^99m^Tc]Tc-GSA	13	No	No	Yes	Yes	Both
van den Esschert et al. [85]	2009	The Netherlands	No	Yes	[^99m^Tc]Tc-Meb	23	No	No	No	Yes	Major
Wakamatsu et al. [86]	2010	Japan	Yes	Yes	[^99m^Tc]Tc-GSA	70	No	No	No	Yes	Minor
Yamao et al. [87]	2019	Japan	No	Yes	[^99m^Tc]Tc-GSA	283	Yes	No	Yes	No	NR^3,4^
Yano et al. [88]	2017	Japan	No	No	[^99m^Tc]Tc-GSA	200	No	Yes	Yes	Yes	Both
Yano et al. [89]	2018	Japan	No	Yes	[^99m^Tc]Tc-GSA	165	Yes	No	No	No	Both
Yoshida et al. [90]	2012	Japan	No	No	[^99m^Tc]Tc-GSA	256	No	Yes	No	Yes	Both
Yoshida et al. [91]	2014	Japan	No	Yes	[^99m^Tc]Tc-GSA	95	No	No	No	Yes	Both^2^
Yumoto et al. [92]	1994	Japan	Yes	No	[^99m^Tc]Tc-GSA	26	Yes	Yes	No	Yes	Both^2^
Yumoto et al. [93]	2010	Japan	No	Yes	[^99m^Tc]Tc-GSA	101	Yes	Yes	No	Yes	Both

*NMT* nuclear medicine test, *[*^*99m*^*Tc]Tc-Meb* ^99m^Tc-labeled mebrofenin, *[*^*99m*^*Tc]Tc-GSA* ^99m^Tc-labeled galactosyl human serum albumin, *[*^*18*^*F]FDG* [^18^F]fluorodeoxyglucose, *[*^*99m*^*Tc]Tc-Sn colloid* ^99m^Tc-labeled sulfur colloid, *[*^*99m*^*Tc]Tc-colloid* ^99m^Tc-labeled colloid, *[*^*99m*^*Tc]Tc-PMT* ^99m^Tc-labeled pyridoxyl-5-methyltryptophan, *[*^*11*^*C]C-Met* L-[methyl-^11^C]-methionine, *[*^*198*^*Au]Au colloid + [*^*99m*^*Tc]Tc-Sn colloid* radioactive colloidal gold and ^99m^Tc-labeled sulfur colloid

^1^Including 88 patients from a prior study from Chapelle et al. [19] to whom they compare the results to and conduct new analyses on these patients

^2^Both major and minor surgery but analyzed separately in the paper

^3^Includes other procedures with the purpose to remove diseased liver parenchyma as well, not only liver resection

^4^The type of liver resection was not defined explicitly

^5^Pancreaticoduodenectomy with reconstructive pancreaticojejunostomy and hepaticojejunostomy, classified as major surgery

^6^Hepatobiliary scintigraphy performed before stage 2 of the ALPPS procedure

^7^Abstract

### Prediction of postoperative mortality

Thirty-seven studies correlated the findings from a preoperative nuclear medicine imaging investigation to postoperative mortality (Table 2). All of the trials involved major intervention (entirely or partly) except for one study in which the extent of liver resection was not described [35]. Postoperative mortality was defined explicitly up front in fifteen trials only. The mortality definitions varied though most used 90-day postoperative mortality. The mortality rate varied considerably (0 to 52%). There were major technical methodological variations across the studies. For example, among the 22 studies investigating [^99m^Tc]Tc-GSA, there were fifteen different ways of calculating the preoperative liver uptake of the tracer (data not shown).

**Table 2:** Overview of trials reporting on the correlation between a preoperative nuclear imaging examination and postoperative mortality

Author	Mortality defined	Mortality event rate	Preset cut off studied	Post hoc cut-off value established	Only descriptive analysis	Mortality vs no mortality comparison	Key diagnostic characteristics reported	Cut-off value predictive in univariate regression analysis	Cut-off value predictive in multivariate regression analysis
Das et al. [23]	Yes	4%	No	Yes	Yes	NR	NR	NR	NR
Dinant et al. [24]	No	11%	No	Yes	No	Yes	Yes	Yes	No
Cieslak et al. [16]	Yes	7%	Yes	No	Yes	NR	NR	NR	NR
Chapelle et al. [19]	Yes	6%	No	Yes^1^	Yes	NR	NR	NR	NR
Chapelle et al. [20]	Yes	0%^2^	Yes^1^	No	Yes	NR	NR	NR	NR
Chiba et al. [18]	No^3^	2%	Yes^1^	No	Yes	NR	NR	NR	NR
Franken et al. [25]	Yes	13%^4^	Yes	No	Yes^5^	NR	NR	NR	NR
Fujioka et al. ≠ [26]	No	7%	No	Yes	Yes	NR	NR	NR	NR
Guiu et al. [27]	Yes	0%	Yes	No	Yes	NR	NR	NR	NR
Hayashi et al. [28]	Yes	6%	Yes	No	No	NR	NR	Yes	NR
Hino et al. [29]	No	6%	No	Yes^6^	Yes	NR	NR	NR	NR
Kaibori et al. [34]	No	2%	No	Yes^1^	Yes	NR	NR	NR	NR
Kim et al. ≠ [39]	No	10%	No	Yes	No	Yes	NR	NR	Yes
Kwon et al. [42]	No	2%	No	Yes	Yes	NR	NR	NR	NR
Kwon et al. [43]	No	2%	No	Yes	Yes	NR	NR	NR	NR
Kwon et al. [44]	No	3%	No	Yes	Yes	NR	NR	NR	NR
Kwon et al. [45]	No	1%	No	Yes	Yes	NR	NR	NR	NR
Kwon et al. ≠ [46]	No	1%	No	Yes^6^	Yes	NR	NR	NR	NR
Namieno et al. [53]	No	0%	Yes	No	Yes	NR	NR	NR	NR
Nishikawa et al. [60]	Yes	42%	No	Yes	No	Yes^7^	NR	Yes	Yes
Nishiyama et al. [61]	No	10%	No	Yes	No	NR	Yes	NR	NR
Okabayashi et al. [63]	Yes	1%	Yes	No	Yes	NR	NR	NR	NR
Olthof et al. [65]	Yes	18%	Yes^8^	Yes^8^	No	NR	Yes^8^	NR	NR
Otsuki et al. [66]	No	27%	No	Yes	No	Yes	NR	NR	NR
Rassam et al. [67]	Yes	11%	Yes	No	No	Yes	NR	NR	NR
Satoh et al. ≠ [68]	No	4%	No	Yes^6^	No	NR	Yes^6^	NR	NR
Serenari et al. [69]	Yes	5%	No	Yes^9^	Yes	NR	NR	NR	NR
Sumiyoshi et al. [75]	Yes	0%	Yes	No	Yes	NR	NR	NR	NR
Takeuchi et al. ≠ [76]	No	8%	No	No	No	Yes^6^	NR	NR	NR
Tanabe et al. [77]	No	52%	No	Yes	No	Yes	NR	NR	NR
Tanaka et al. [79]	No	2%^10^	Yes	No	Yes	NR	NR	NR	NR
Truant et al. [82]	No	20%	No	No	Yes	NR	NR	NR	NR
Truant et al. [83]	No	14%^11^	No	No	Yes	NR	NR	NR	NR
Yamao et al. [87]	Yes	25%^12^	No	No	No	NR	NR	No	NR
Yano et al. [89]	Yes	18%^13^	No	Yes	No	Yes^7^	NR	NR	No
Yumoto et al. [92]	No	12%	No	Yes	Yes	NR	NR	NR	NR
Yumoto et al. [93]	No	3%	No	Yes	Yes	NR	NR	NR	NR

Papers with mortality as part of a composite endpoint are marked with a “≠”; in those cases, the data are reported for overall complications in which mortality is included unless otherwise stated

*NR* not reported

^1^The cut-off value established for the prediction of postoperative LF is also used for the outcome mortality

^2^No patients in the interventional group died postoperatively, but in the 88 patients in the prior study by Chapelle et al. the mortality rate was 6%, also reported in the table above

^3^Two patients died due to LF during the hospital stay. The NMT uptake values are reported for these two patients

^4^In the overall population including the cohort from 2000 to 2015 and the cohort from 2016 to 2019

^5^Comparison of outcomes in the cohort from 2000 to 2015 to those in the cohort from 2016 to 2019 applying different cut-off values for the function of the FLR

^6^Cut-off value, diagnostic characteristics, or mortality vs. no mortality for overall complications or poor outcome, but separate uptake values reported for patients who died postoperatively

^7^They perform a log-rank test to compare survival rates in patients with liver uptake function above and below a certain level

^8^Cut-off value for postoperative LF

^9^AUC value only investigated for patients developing LF-related mortality (16%)

^10^Only in 98 patients who underwent hemi-hepatectomy did the authors evaluate mortality and LF. Therefore, the mortality event rate was 2/98 (2%)

^11^Postoperative clinical outcomes were only reported in the seven patients who underwent ALPPS. One of the seven patients died due to LF postoperatively

^12^5-year overall mortality rate based on the reported 5-year overall survival rates of 55.2% in one group (*n* = 24) and 77.3% in another group (*n* = 259)

^13^5-year overall mortality rate based on the reported 5-year overall survival rate of 82%. The 3-year tumor-free survival rate was 41%

Most studies retrospectively reported the nuclear medicine investigation results among patients with a fatal outcome versus those with a non-fatal outcome or described the proportion of patients with a fatal outcome below or above a defined cut-off value. A minority of the studies provided clinically relevant prognostic information in the form of preoperative nuclear medicine results in patients with fatal versus nonfatal outcomes, analyzed diagnostic test accuracy characteristics, or assessed predictive values in univariate and multivariate analyses (Table 2). Thirty-three studies described a preoperative cut-off value for the prediction of postoperative mortality or mortality as part of a composite outcome. Twelve of these studies used a predetermined cut-off value. Five papers (of which two reports had mortality as part of a composite endpoint) compared nuclear medicine results in patients with fatal versus non-fatal outcomes. All studies found significant differences in the nuclear medicine investigation results between patients with a fatal outcome and those with a non-fatal outcome. Two papers compared survival rates in patients with liver function uptake values above or below a certain level (reported in the same column). Nishikawa et al. [60] found a significant difference between survival rates in patients above and below a certain cut-off, whereas Yano et al. [89] did not. Moreover, Rassam et al. [67] did not find a significant association between 90-day mortality and the preoperative FLR function or uptake rate of [^99m^Tc]Tc-mebrofenin. Four papers retrospectively assessed the diagnostic test accuracy of the cut-off determined based on the observations from their study. Satoh et al. [68] analyzed the diagnostic characteristics of the cut-off value in regard to predicting overall complications in which mortality was included, whereas Dinant et al. [24] and Nishiyama et al. [61] analyzed the diagnostic characteristics of the cut-off value for the prediction of LF-related mortality. The positive predictive values were modest (50 to 71%), whereas the negative predictive values were excellent (98 to 100%). Dinant et al. [24] reported a sensitivity of 75% and specificity of 93% for predicting LF-related mortality. Olthof et al. [65] found that the FLR function had an AUC of 0.70 for the prediction of liver failure-related mortality.

Six papers assessed the predictive value of the preoperative nuclear medicine test for postoperative mortality or a composite outcome in univariate and/or multivariate regression analysis. Dinant et al. [24] found that future remnant (FR) uptake determined by [^99m^Tc]Tc-mebrofenin liver imaging had significant predictive value for LF-related mortality but not all-cause mortality in univariate analysis but not multivariate analysis. Hayashi et al. [28] showed that the FR function determined from [^99m^Tc]Tc-GSA imaging was a significant predictor of postoperative mortality (odds ratio (OR) 8.8, *p* = 0.008) in univariate analysis; no multivariate analysis was performed. Kim et al. [39] found LHL15 to be a significant predictor of overall complications, including mortality, in multivariate analysis. Nishikawa et al. [60] found the GSA index to be a significant predictor of recurrence-free survival and overall survival in univariate analysis and documented a significant predictive value of the GSA index for recurrence-free survival in multivariate cox regression analysis (hazard ratio (HR) 2.4, *p* < 0.001). However, Yano et al. [89] found that the GSA-R_max_ parameters were not associated with overall survival or tumor-free survival in multivariate cox regression analysis, and Yamao et al. [87] did not find a significant association between LHL15 and overall survival in univariate cox regression analysis. Several other studies claimed to document the predictive value of the imaging tests evaluated, but the findings were not supported by statistical analyses.

### Prediction of postoperative LF

Fifty-two studies reported on LF alone or as part of a composite outcome (Table 3). Fifty (96%) of the trials involved major surgery (entirely or partly). Among the forty-four studies reporting on the definition of postoperative LF, the definition varied considerably (data not shown). The postprocedural LF rate ranged from 0 to 86% (Table 4). Most trials (*n* = 35) were performed with [^99m^Tc]Tc-GSA, of which more than 25 different measures of liver function were applied (data no shown). Twenty-one studies analyzed a predetermined cut-off value, and nine studies did not report a cut-off value. The remaining studies established a post hoc (data-driven) cut-off value, and some reported both.

**Table 3:** Overview of the studies reporting on the correlation between a preoperative nuclear imaging examination and the postoperative outcome liver failure

Author	Liver failure defined	LF event rate	Preset cut-off value studied	Post hoc cut-off value established	Only descriptive analysis	Liver failure vs. no liver failure comparison	Key diagnostic characteristics reported	Cut-off value predictive in univariate regression analysis	Cut-off value predictive in multivariate regression analysis
Cieslak et al. [16]	Yes	2%	Yes	No	Yes	NR	NR	NR	NR
Cieslak et al. ≠ [17]	No	11%^1^	Yes	No^2^	Yes	NR	NR	NR	NR
Chapelle et al. [19]	Yes	14%	No	Yes	No	Yes	Yes	Yes	Yes
Chapelle et al. [20]	Yes	1%^3^	Yes	No	Yes	NR	NR	NR	NR
Chapelle et al. [21]	Yes	15%	Yes	No	No	Yes	Yes	NR	Yes
Chiba et al. [18]	Yes	6%^4^	Yes	No	No	NR	NR	Yes	Yes
Cho et al. [22]	Yes	5%	No	Yes	No	Yes	Yes	Yes	Yes
de Graaf et al. [10]	Yes	16%	No	Yes	No	Yes	Yes	NR	NR
Dinant et al. [24]	Yes	13%	No	Yes	No	Yes	Yes	Yes	Yes
Franken et al. [25]	Yes	15%^5^	Yes	No	Yes^6^	NR	NR	NR	NR
Guiu et al. [27]	Yes	0%	Yes	No	Yes	NR	NR	NR	NR
Hayashi et al. [28]	Yes	8%	Yes	No	No	NR	NR	Yes	NR
Hino et al. ≠ [29]^7^	Yes	4%	No	Yes	Yes	NR	NR	NR	NR
Hirai et al. [30]	Yes	23%	No	Yes	No	Yes	Yes	NR	NR
Hwang et al. ≠ [31]	No	9%	No	No	No	Yes	NR	NR	NR
Kaibori et al. [34]	Yes	8%	No	Yes	No	Yes	Yes	Yes	Yes
Kamohara et al. ≠ [35]	Yes	NR (30% overall)	Yes	No	Yes	NR	NR	NR	NR
Kato et al. ≠ [36]	Yes	33%^8^	No	Yes	No	Yes	Yes	NR	Yes
Katsuramaki et al. ≠ [37]^7^	Yes	3%	No	Yes	Yes	NR	NR	NR	NR
Kawamura et al. [38]	No	0%	No	No	Yes	NR	NR	NR	NR
Kim et al. ≠ [39]	No	10%	No	Yes	No	Yes	NR	NR	Yes
Kokudo et al. [40]	Yes^9^	13%	No	Yes	No	Yes	NR	NR	Yes
Kwon et al. [45]	Yes	5%	No	Yes	Yes	NR	NR	NR	NR
Mao et al. [48]	Yes^10^	13%	Yes	Yes	No	NR	Yes	NR	NR
Mitsumori et al. [49]	No^11^	10%	Yes	No	Yes	NR	NR	NR	NR
Nakamura et al. [50]	Yes	17%	No	Yes	No	Yes	NR	NR	Yes
Nakano et al. ≠ [51]	Yes	6%	Yes	No	No	Yes	NR	NR	No
Nanashima et al. ≠ [54]^7^	Yes	6%	No	Yes	No	Yes	NR	NR	NR
Nanashima et al. ≠ [55]	Yes	6%	No	Yes	No	Yes	NR	NR	No
Nanashima et al. ≠ [56]	Yes	8%	Yes (later group)	Yes (earlier group)	No	Yes	NR	Yes	No
Nanashima et al. ≠ [57]	Yes	5%	No	Yes	No	Yes	NR	NR	Yes (LF only)
Nanashima et al. [58]	No	4%	Yes	No	Yes	NR	NR	NR	NR
Nanashima et al. [59]	Yes	1%	No	No	No	Yes	NR	NR	NR
Nitta et al. [62]	Yes	18%	Yes	Yes	No	Yes	Yes	NR	Yes
Okabayashi et al. [63]	Yes	10%^12^	Yes	No	Yes	NR	NR	NR	NR
Okabe et al. [64]	Yes	PLD 7% LF 2%	No	Yes (PLD)	No	Yes (PLD)	Yes (PLD)	NR	Yes (PLD)
Olthof et al. [65]	Yes	23%	Yes	Yes	No	Yes	Yes	Yes	Yes
Rassam et al. ≠[67]^7^	Yes	10% (LF only)	Yes	No	No	Yes	NR	NR	NR
Satoh et al. ≠ [68]^7^	Yes	9%	No	Yes	No	NR	Yes	NR	NR
Serenari et al. [69]	Yes	25%^13^	No	Yes	No	Yes	Yes	NR	NR
Serenari et al. [70]	Yes	33%	No	No	No	Yes	NR	NR	NR
Sugai et al. ≠ [73]	No	18%	No	No	No	Yes	NR	NR	NR
Sumiyoshi et al. [74]	Yes	7%^14^	Yes	No	No	Yes	NR	NR	NR
Sumiyoshi et al. [75]	Yes	0%	Yes	No	Yes	NR	NR	NR	NR
Tanaka et al. [79]	Yes	41%^15^	Yes	No	No	NR	NR	Yes	NR
Tanoue et al. [81]	Yes	15%	No	No^16^	No	Yes	NR	NR	NR
Truant et al. [82]	Yes	80%^17^	No	No	Yes	NR	NR	NR	NR
Truant et al. [83]	Yes	86%^18^	No	No	Yes	NR	NR	NR	NR
Yano et al. [88]	Yes	21%	No	No^16^	No	Yes	NR	NR	NR
Yoshida et al. [90]	Yes	6%	No	Yes	No	Yes	Yes	NR	Yes
Yumoto et al. [92]	No	19%	No	Yes	Yes	NR	NR	NR	NR
Yumoto et al. [93]	Yes^9^	17%	No	Yes	No	Yes	Yes	NR	NR

Papers with liver failure as part of a composite endpoint are marked with a “≠”; in those cases, the data are reported for overall complications in which liver failure is included unless otherwise stated

*LF* liver failure, *PLD* postoperative liver dysfunction

^1^Including three patients who died due to LF, but they only looked at the NMT in regard to developing LF postoperatively

^2^Not for the prediction of LF, but they establish a cut-off for the prediction of reaching a sufficient function of the FLR after PVE

^3^1% of patients in the interventional group developed LF postoperatively, but in the 88 patients in the prior study by Chapelle et al., the liver failure rate was 14%, also reported in the table above

^4^Including two patients who died due to LF. Their primary endpoint was LF, but they also provided uptake values for the patients who died due to LF, also reported in mortality table

^5^In the overall population including the cohort from 2000 to 2015 and the cohort from 2016 to 2019

^6^Comparison of outcomes in the cohort from 2000 to 2015 to those in the cohort from 2016 to 2019 applying different cut-off values for the function of the FLR

^7^Cut-off value, diagnostic characteristics, predictive value, or LF vs. no LF for overall complications or poor outcome, but separate uptake values for patients who developed LF postoperatively

^8^Including 3 patients who died due to postoperative LF

^9^Signs of postoperative LF

^10^A Child Score > 9 is considered a high risk for developing LF

^11^Specifically described as hepatic insufficiency not LF

^12^Meeting the criteria for LF based on at least one of three definitions

^13^Including one patient who developed LF after stage 1 of the ALPPS procedure, and the other four patients developed LF after stage 2 of the ALPPS procedure

^14^LF-related Clavien-Dindo grade III complication

^15^Only in 98 patients who underwent hemi-hepatectomy did the authors evaluate mortality and LF. Therefore, the liver failure event rate was 40/98 (41%)

^16^Not for the prediction of LF, but they establish a cut-off for the prediction of postoperative long-term ascites

^17^Including one patient who developed LF and died postoperatively

^18^Postoperative clinical outcomes were only reported in the seven patients who underwent ALPPS. Six of the seven patients developed LF, five after stage 2 of the ALPPS procedure and one postoperatively who also died. The other five recovered postoperatively

**Table 4:** Details of the diagnostic characteristics, descriptive values, and predictive value of the preoperative nuclear imaging examinations and postoperative liver failure

Author	Cut-off value (variable, value and unit)	Liver failure vs. no liver failure (mean or median, ***p*** value)	Key diagnostic characteristics	Univariate predictive regression analysis (impact, ***p*** value)	Multivariate predictive regression analysis (impact, ***p*** value)
Chapelle et al. [19]	eFLRF, 2.3 (%/min/m^2^)	2.2 vs. 4.7 (*p* < 0.001)	Sens 92%, spec 98%, PPV 92%, NPV 99%	For eFLRF < 2.3: OR 836 (*p* < 0.001)	NR (*p* = 0.001)
	TLF, NR (%/min)^1^	5.0 vs. 6.2 (*p* = 0.020)	NR	NR (*p* = 0.027)	NR (NS)
Chapelle et al. [21]	eFLRF, 2.3 (%/min/m^2^)	3.3 vs. 8.4 (*p* < 0.001)	AUC 0.843	NR (NR)	OR 0.35 (*p* = 0.002)
	HBS^BSA^, NR (%/min)^1^	5.5 vs. 6.1 (*p* = 0.057)	AUC 0.652	NR (NR)	NR (NR)
Chiba et al. [18]	Remnant liver LU 15, 13	NR (NR)	NR	Cont. LU 15: OR 0.92 (NS)^2^ rLU 15 < 13: OR 81.8 (*p* < 0.001)^2^	Cont.: NR (NR)^2^ rLU 15 < 13: OR 67.7 (*p* < 0.001)^2^
	Total liver LU 15, NR	NR (NR)	NR	OR 0.97 (NS)	NR (NR)
	LHL15, NR	NR (NR)	NR	OR 0.04 (NS)	NR (NR)
	HH15, NR	NR (NR)	NR	OR 0.07 (NS)	NR (NR)
Cho et al. [22]	SUV_mean_, 2.4	2.1 vs. 2.3 (NS)	Sens 100%, spec 32%, PPV 7%, NPV 100%	For SUV_mean_ ≤ 2.4: OR 7.0 (NS)	NR (NR)
	TLG_r_, 625.6	1067 vs. 1491 (NS)	Sens 57%, spec 97%, PPV 44%, NPV 98%	For TLG_r_ ≤ 625.6: OR 36.5 (*p* < 0.001)	For TLG_r_ ≤625.6: OR 82.9 (*p* < 0.001)
de Graaf et al. [10]	FRL-F, 2.69 (%/min/m^2^)	2.2 vs. 4.3 (*p* = 0.001)	Sens 89%, spec 87%, PPV 57%, NPV 98%	NR (NR)	NR (NR)
Dinant et al. [24]	FRL-uptake, 2.5 (%/min/BSA)	2.3 vs. 4.3 (*p* = 0.003)	Sens 83%, spec 90%, PPV 56%, NPV 97%	NR (*p* = 0.01)	OR 4.0 (*p* = 0.03)
Hayashi et al. [28]	Marginal FR function, NR (%)	NR (NR)	NR	For marginal FR function vs. safe FR function: OR 11.0 (*p* = 0.001)	NR (NR)
Hirai et al. [30]	[^99m^Tc]Tc-GSA uptake, 25% of non-embolized liver	10.0% vs. 25.8% (*p* = 0.02)^3^	Sens 50%, spec 94%	NR (NR)	NR (NR)
Hwang et al. [31]	Predicted, residual [^99m^Tc]Tc-GSA-clearance, NR (mL/min)	90 vs. 321 (*p* < 0.005)	NR	NR (NR)	NR (NR)
	Total [^99m^Tc]Tc-GSA-clearance, NR (mL/min)	315 vs. 567 (NS)	NR	NR (NR)	NR (NR)
Kaibori et al. [34]	HA/GSA-Rmax ratio, 500 (mg min/dl)	NR (*p* < 0.0001)^4^	Sens 88%, spec 92%, PPV 50%, NPV 99%	For HA/GSA-Rmax ratio ≥ 500: OR 21.5 (*p* < 0.0001)	For HA/GSA-Rmax ratio ≥ 500: OR 23.6 (*p* = 0.0138)
	GSA-Rmax, 0.475 (mg/min)	NR (*p* = 0.0016)^4^	NR	For GSA-Rmax < 0.475: OR 8.1 (*p* = 0.0066)	For GSA-Rmax < 0.475: OR 0.2 (NS)
	Type IV collagen 7S/GSA-Rmax, 15 (mg min/dL)	NR (*p* < 0.0001)^4^	NR	For Type IV collagen 7S/GSA-Rmax ≥ 15: OR 18.1 (*p* = 0.0056)	For Type IV collagen 7S/GSA-Rmax ≥ 15: OR 7.7 (NS)
Kato et al. [36]	LHL15, NR	0.90 vs. 0.92 (*p* = 0.006)	AUC 0.68	NR (NR)	Per 0.1-unit increment: OR 0.24 (*p* = 0.046)
	HH15, NR	0.66 vs. 0.63 (NS)	NR	NR (NR)	NR (NR)
	GSA-Rmax, NR	0.61 vs. 0.71 (*p* = 0.03)	AUC 0.62	NR (NR)	Per 0.1-unit increment: OR 0.81 (NS)
	rERL-GSA, NR	0.53 vs. 0.60 (NS)	AUC 0.62	NR (NR)	NR (NR)
	ERL-LHL15, NR	0.48 vs. 0.55 (*p* = 0.04)	AUC 0.64	NR (NR)	NR (NR)
	ERL-Rmax, NR^5^	0.33 vs. 0.41 (*p* = 0.004)	AUC 0.66	NR (NR)	NR (NR)
Kim et al. ≠ [39]	LHL15, 0.91	0.85 vs. 0.94.(*p* < 0.0001)	NR	NR (NR)	NR (*p* < 0.0001)
Kokudo et al. [40]^6^	R_0_-remnant, 0.16 (μmoles)	0.015 vs. 0.024 (*p* = 0.011)	NR	NR (NR)	Per 0.01 μmoles increment: HR 0.82 (*p* = 0.022)
	LHL15, NR	0.89 vs. 0.93 (*p* = 0.025)	NR	NR (NR)	NR (NS)
	HH15, NR	0.58 vs. 0.52 (NS)	NR	NR (NR)	NR (NR)
	R_0_, NR (μmole)	0.14 vs. 0.18 (*p* = 0.038)	NR	NR (NR)	NR (NS)
	[R]_0_, NR (μM)	0.63 vs. 0.70 (NS)	NR	NR (NR)	NR (NR)
Mao et al. [48]	Uptake index, 0.9^7^	NR (NR)	Sens 100%, spec 92%	NR (NR)	NR (NR)
Nakamura et al. [50]	LHL15, NR	0.875 vs. 0.903 (*p* = 0.015)	NR	NR (NR)	OR 1.32 (NS)
	Remnant LHL15, 0.755	0.739 vs. 0.791 (*p* = 0.009)	NR	NR (NR)	OR 0.03 (*p* = 0.023)
Nakano et al. ≠ [51]	GSA-Rmax, 0.60	NR (NS)	NR	NR (NR)	NR (NS)
Nanashima et al. ≠ [54]	LHL15, 0.875	NR (*p* < 0.001)^4^	NR	NR (NR)	NR (NR)
Nanashima et al. ≠ [55]	LHL15, 0.85	91.1 vs. 93.1 (*p* = 0.014)	NR	NR (NR)	For LHL15 < 0.85 vs. ≥ 0.85: OR 1.4 (NS)
Nanashima et al. ≠ [56]	LHL15, 0.85	92.2 vs. 95.2 (*p* = 0.021)	NR	For LHL15 < 0.85 vs. ≥ 0.85: OR 5.1 (*p* = 0.022)	For LHL15 < 0.85 vs. ≥ 0.85: OR 3.8 (NS)
Nanashima et al. ≠ [57]	LHL15, 0.90	0.89 vs. 0.92 (*p* = 0.015)	NR	NR (NR)	For LHL15 < 0.90 vs. ≥ 0.90: OR 2.7 (NS) (LF)
	HH15, 0.60 (*Y* values of regression equation for LF only, 7.2)	0.61 vs. 0.58 (*p* < 0.01)	NR	NR (NR)	For HH15 ≥ 0.60 vs. < 0.60: OR 3.3 (*p* = 0.045) (LF)
	LHL15/HH15, 1.60	1.53 vs. 1.64 (*p* < 0.01)	NR	NR (NR)	For LHL15/HH15 < 1.60 vs. ≥ 1.60: OR 0.9 (NS) (LF)
Nanashima et al. [59]	LHL15/HH15, NR	1.52 vs. 1.64 (NS)	NR	NR (NR)	NR (NR)
	LHL15 minus HH15, NR	31.0 vs. 34.6 (NS)	NR	NR (NR)	NR (NR)
Nitta et al. [62]	Fvo-0%, marginal function^8^	NR (*p* = 0.031)^4^	C-index ≈ 0.67	NR (NR)	NR (NS)
	Fvo-40%, marginal function^8^	NR (*p* = 0.031)^4^	C-index = 0.737	NR (NR)	For marginal vs. safe function: OR 9.0 (*p* = 0.002)
	Fvo-100%, marginal function^8^	NR (*p* = 0.013)^4^	C-index ≈ 0.65	NR (NR)	NR (NS)
Okabe et al. [64]	LHL15, 0.93^9^	0.92 vs. 0.93 (*p* = 0.0027)	Sens 88%, spec 96%	NR (NR)	For LHL15 ≤ 0.93: OR 7.4 (*p* = 0.0082)
	HH15, NR^9^	0.66 vs. 0.58 (*p* = 0.0041)	NR	NR (NR)	NR (NR)
Olthof et al. [65]	Total liver function, NR (%/min)	14.6 vs. 16.2 (*p* = 0.41)	NR	NR (NR)	NR (NR)
	FLR function, NR (%)	44.7 vs. 63.4 (*p* < 0.01)	AUC 0.68	NR (NR)	NR (NR)
	FLR function, 8.5 (%/min) (new cut-off)	5.6 vs. 8.7 (*p* < 0.01)	PPV 36%, NPV 91% (new cut-off)^10^ AUC 0.69	For FLR function < 8.5: OR 5.4 (*p* < 0.01)	For FLR function < 8.5: OR 4.1 (*p* < 0.01)
	sFLR function, 2.7 (%/min/m^2^) (predefined cut-off)	3.1 vs. 4.7 (*p* < 0.01)	PPV 38%, NPV 82% (pre-defined cut-off)^10^ AUC 0.68	NR (NR)	NR (NR)
Rassam et al. ≠ [67]	FRL function, 2.7 (%/min/m^2^) (predefined cut-off)	NR (NS)	NR	NR (NR)	NR (NR)
	MUR NR (%/min)	NR (NS)	NR	NR (NR)	NR (NR)
Satoh et al. ≠ [68]	PRI, 0.38	NR (NR)	PPV 71%, NPV 100%	NR (NR)	NR (NR)
Serenari et al. [69]	FLR-C, 34.5 (%)	30 vs. 41 (*p* = 0.011)	Sens 100%, spec 82%, PPV 50%, NPV 100%	NR (NR)	NR (NR)
	FLR-F, 1.69 (%/min/m^2^)	0.94 vs. 2.07 (*p* = 0.011)	Sens 100%, spec 75%, PPV 50%, NPV 100%	NR (NR)	NR (NR)
	HIBA-i, 14.94 (%)	12.86 vs. 23.29 (*p* = 0.001)	Sens 100%, spec 94%, PPV 80%, NPV 100%	NR (NR)	NR (NR)
Serenari et al. [70]	FRL-F, NR (%/min/m^2^)	1.72 vs. 4.02 (NR)	NR	NR (NR)	NR (NR)
Sugai et al. ≠ [73]	LUR/ LUR response rate, NR (%/%)	NR (NS) / NR (NS)^11^	NR	NR (NR)	NR (NR)
	FV / FV response rate, NR (cm^3^/%)	NR (NS)/NR (NS)^11^	NR	NR (NR)	NR (NR)
	LUD/LUD response rate, NR (%/cm^3^/%)	0.035 vs. 0.064 (*p* < 0.05)/− 8.9 vs. 22.2 (*p* < 0.01)^11^	NR	NR (NR)	NR (NR)
Sumiyoshi et al. ≠ [74]	remKGSA, 0.05	NR (*p* < 0.02)	NR	NR (NR)	NR (NR)
	KGSA, NR	NR (NS)	NR	NR (NR)	NR (NR)
Tanaka et al. [79]	LHL15, NR	NR (NR)	NR	NR (*p* < 0.001)	NR (NR)
	GSA-index, NR	NR (NR)	NR	NR (*p* = 0.001)	NR (NR)
	Remnant VLmg, NR	NR (NR)	NR	NR (*p* = 0.001)	NR (NR)
Tanoue et al. [81]	GSA-Rmax, NR (mg/min)	0.479 vs. 0.501 (NS)	NR	NR (NR)	NR (NR)
	rGSA-Rmax, NR (mg/min)	0.319 vs. 0.374 (*p* = 0.032)	NR	NR (NR)	NR (NR)
	Difference between GSA-Rmax and rGSA-Rmax, NR (mg/min)	0.160 vs. 0.127 (NS)	NR	NR (NR)	NR (NR)
	rGSA-Rmax/GSA-Rmax, NR	0.692 vs. 0.756 (*p* = 0.042)	NR	NR (NR)	NR (NR)
Yano et al. [88]	GSA-Rmax, NR (mg/min)	0.432 vs. 0.453 (NS)	NR	NR (NR)	NR (NR)
Yoshida et al. [90]	rLUV_(BSA)_, 27.0%/BSA	23.0 vs 33.6 (*p* < 0.001)^12^	Sens 91%, spec 81%, PPV 31%, NPV 99%	NR (NR)	NR (*p* < 0.001)
	HH15, NR	0.64 vs. 0.60 (*p* < 0.05)^12^	NR	NR (NR)	NR (NS)
	LHL15, NR	0.90 vs. 0.91 (NS)^12^	NR	NR (NR)	NR (NR)
	% remnant LF, NR (%)	60.9 vs. 75.3 (*p* < 0.001)^12^	NR	NR (NR)	NR (NS)
Yumoto et al. [93]	R0-remnant, 100 (nmol/liver)	62.1 vs. 122.2 (*p* < 0.001)	AUC 0.97	NR (NR)	NR (NR)
	[R]0, NR (nmol/l)	412 vs. 551 (*p* = 0.045)	AUC 0.80	NR (NR)	NR (NR)
	R0, NR (nmol/liver)	149.8 vs. 211.2 (*p* = 0.047)	NR	NR (NR)	NR (NR)
	LHL15, NR	0.79 vs. 0.87 (*p* = 0.035)	AUC 0.74	NR (NR)	NR (NR)

Papers with liver failure as part of a composite endpoint are marked with a “≠”; in those cases, the data are reported for overall complications in which liver failure is included unless otherwise stated (marked LF). For the diagnostic characteristics, sens, spec, NPV, and PPV were reported if available; otherwise, AUC was reported if available

*eFLRF* estimated future remnant liver function, *NR* not reported, *Sens* sensitivity, *Spec* specificity, *PPV* positive predictive value, *NPV* negative predictive value, *TLF* total liver function, *HBS*^*BSA*^ global liver function ([^99m^Tc]Tc-mebrofenin hepatobiliary scintigraphy clearance divided by body surface area), *SUV*_*mean*_ mean standardized uptake value, *TLG*_*r*_ total glycolysis of the remnant liver, *LU 15* the cumulative liver uptake of the tracer 15 to 16 min after injection of the tracer, *Cont*. continuous, *LHL15* [^99m^Tc]Tc-GSA receptor index, *R*_*0*_*-remnant* total hepatic asialoglycoprotein receptor amount in the future remnant liver, *HH15* [^99m^Tc]Tc-GSA clearance index, *FRL-F* future remnant liver uptake function, *FRL* future remnant liver, *FR* future remnant, *HA/GSA-Rmax ratio* the ratio of serum hyaluronic acid to the maximum removal rate of [^99m^Tc]Tc-GSA, *GSA-Rmax* the maximum removal rate of [^99m^Tc]Tc-GSA, *Type IV collagen 7S/GSA-Rmax* the ratio of type IV collagen 7S to the maximum removal rate of [^99m^Tc]Tc-GSA, *rERL-GSA* ratio of preoperatively estimated remnant liver counts (ERL) to total liver counts, *ERL-LHL15* hepatic uptake ratio (LHL15) of estimated remnant liver, *ERL-Rmax* maximal removal rate of estimated remnant liver counts, *R*_*0*_ total hepatic asialoglycoprotein receptor amount, *[R]*_*0*_ hepatic asialoglycoprotein receptor concentration, *LHL15/HH15* GSA index, *cICGR15* converted indocyanine green retention rate at 15 min calculated from HH15, LHL15, and hyaluronic acid level, *Fvo* presumed function of the veno-occlusive region of the liver, *FLR* future liver remnant, *sFLR* standardized future liver remnant, *MUR* the mebrofenin uptake rate, *PRI* predictive residual index, *FRL-C* percentage of counts within the future remnant liver, *HIBA-i* the HIBA-index (the proportion of radionuclide accumulated in the future remnant liver), *LUR* liver uptake ratio, *FV* functional liver volume, *LUD* liver uptake density, *KGSA* the estimated indocyanine green plasma clearance rate using the hepatic uptake ratio of [^99m^Tc]Tc-GSA (LHL15), *remKGSA* KGSA of the future remnant liver, *Remnant VLmg* amount of [^99m^Tc]Tc-GSA accumulation in the remnant liver, *rGSA-Rmax* the maximum removal rate of [^99m^Tc]Tc-GSA in the remnant liver, *rLUV*_*(BSA)*_ liver uptake value of the remnant liver corrected for body surface area, *% remnant LF* the relative residual liver function, *(LF)* for predicting LF exclusively

^1^TLF and HBS^BSA^ corresponds to the same global liver function estimate from [^99m^Tc]Tc-mebrofenin hepatobiliary scintigraphy, but in the two papers they are named differently

^2^They report both the predictive value of the continuous remnant LU 15 value and the predictive value of the cut-off of remnant LU 15 < 13. The latter resulting in a significant predictive value

^3^Before PVE

^4^Not a comparison of liver function uptake value between patients with liver failure or without but a comparison of LF rate in patients with liver uptake value above and below a certain value (cut-off)

^5^In addition to these GSA-parameters of liver function, they also report the diagnostic characteristics, cut-off value, predictive value in univariate and multivariate analysis of the actual remnant liver (ARL) GSA-parameters (rARL-GSA, ARL-LHL15, and ARL-Rmax) with updated delineations of the FLR based on the pre- and postoperative CT-scans. These are not shown in this table but can be found in the paper

^6^For “Signs of postoperative Liver Failure”

^7^For predicting patients with a postoperative Child score of ≥ 9, which corresponds to a high risk of liver failure

^8^They are all based on the liver uptake ratio of [^99m^Tc]Tc-GSA

^9^Reported here for patients with a % FLR (volume-based) of 35–60%, further details are provided for patients > 60% FLR in the original paper

^10^In the whole patient population, not just in the patients with bilirubin level below 50 μmol/L at the time of hepatobiliary scintigraphy. For these patients the PPV 41%, NPV 94%

^11^Post percutaneous transhepatic portal embolization (PTPE) values listed here. The pre-PTPE values are also reported in the paper for the patient with and without complications, but not listed here

^12^Non-preserved vs. preserved liver function on postoperative day 5; non-preserved referring to moderate-severe hepatic dysfunction on postoperative day 5 and preserved referring to no or mild hepatic dysfunction on postoperative day 5

A large number of trials (*n* = 35) provided detailed diagnostic or predictive data (Table 4). Twenty-six trials compared the results of radionuclide imaging in patients with or without liver failure for one or more imaging variables. Most trials showed significant differences among patients with and without liver failure for at least one of the liver function parameters. Sixteen studies analyzed the diagnostic characteristics of the cut-off values in predicting postoperative LF or overall complications. In general, the sensitivity and specificity of the cut-off values varied considerably from 50 to 100% and from 32 to 98%, respectively. The positive predictive values of the cut-offs varied considerably as well (7 to 92%), whereas the negative predictive values were consistently high (82 to 100%).

Twenty trials analyzed the predictive value of the nuclear medicine test in predicting postoperative LF or a composite outcome including LF in univariate and/or multivariate regression analyses, and some trials presented up to six variables. All studies showed significant outcome in univariate regression analysis with at least one nuclear medicine variable. Multivariate regression analyses were performed in eighteen trials, fifteen of which showed that a nuclear medicine liver function test was a significant independent predictor of postoperative LF (Table 4). Twelve of these trials presented the predictive impact with an OR or HR. Eleven of these studies tested other variables as well, and only six of these studies [21, 22, 36, 57, 64, 65] found that other variables (e.g., operation time, extent of hepatectomy, blood loss volume, and aspartate aminotransferase to platelet ratio index) had significant predictive value for predicting LF.

### Historical comparisons

Five studies reported historical comparisons on the outcome rate of LF and mortality in the period before and after implementation of nuclear imaging as a preoperative examination in patients undergoing liver surgery [16, 20, 25, 56, 58]. Overall, the historical comparisons involving [^99m^Tc]Tc-mebrofenin found that implementation of nuclear imaging in the preoperative assessment resulted in lower mortality and liver failure rates [16, 20, 25], whereas the historical comparisons on the use of [^99m^Tc]Tc-GSA did not result in a significant decrease in the number of liver failure cases in the period after implementation of [^99m^Tc]Tc-GSA liver scintigraphy [56, 58].

## Discussion

To the best of our knowledge, this is the first systematic review investigating the value of preprocedural nuclear imaging examinations for the prediction of postprocedural mortality and LF in patients undergoing localized, liver-directed interventions. This review demonstrated great technical heterogeneity, e.g., in terms of tracers, nuclear imaging uptake calculations, and outcome definitions. Most trials were retrospective and explored test-derived cut-off values rather than evaluating the clinical validity of predetermined variables and cut-off values. However, a few studies investigated predetermined cut-off values and confirmed the clinical utility of these for the preoperative nuclear imaging examination both in terms of producing low LF and mortality rates. In addition, a notable number of trials reported significant predictive values of the nuclear medicine imaging test in multivariate analyses for the prediction of LF, which favors further efforts to identify the clinical utility of these tests in a prospective setting.

The most promising and well-investigated nuclear medicine imaging tracers for the prediction of postoperative clinical outcome were [^99m^Tc]Tc-GSA and [^99m^Tc]Tc-mebrofenin. Despite the fact that a large proportion of these trials showed interesting diagnostic properties and excellent predictive values, the methodology was not optimal in most cases. The definitions of liver failure and mortality differed across the studies without consensus on predefined, clinically relevant liver failure and mortality definitions. This may ultimately affect the outcome of the individual analyses and complicate the ability to properly compare the tracers in the studies in terms of their predictive value. Furthermore, the majority of the studies were retrospective and had an exploratory approach, and the diagnostic properties of the nuclear imaging techniques were defined based on the actual observations. A few papers applied a predetermined cut-off value in a prospective setting and investigated outcomes among patients with uptake values above or below that cut-off. Thus, there were only a few clinical utility studies. However, the studies applying a predetermined cut-off value in a prospective setting validated the cut-off and proved that the cut-off value was able to safely determine which patients could undergo hepatic resection with resulting low mortality and LF rates [20, 63], thereby underscoring the importance of preoperative nuclear imaging in patients undergoing liver resection. A few studies investigated other tracers; the limited number of data makes it difficult to identify advantages or disadvantages over [^99m^Tc]Tc-GSA and [^99m^Tc]Tc-mebrofenin.

The major differences between [^99m^Tc]Tc-GSA and [^99m^Tc]Tc-mebrofenin exists in the uptake and excretion of these tracers. The uptake of [^99m^Tc]Tc-GSA follows receptor-mediated endocytosis by attachment to asialoglycoprotein receptors on the functioning hepatocytes. The tracer is then transferred to the lysosomes for degradation [94]. As the only uptake site for [^99m^Tc]Tc-GSA is in the liver, imaging with [^99m^Tc]Tc-GSA offers a good representation of the functioning hepatocytes. In comparison, [^99m^Tc]Tc-mebrofenin is transported to the liver predominantly bound to albumin after which it is taken up by the functioning hepatocytes and excreted unmetabolized into the bile system. As a result, imaging with [^99m^Tc]Tc-mebrofenin offers visualization of the liver’s uptake and excretion function including the biliary tract system [94]. However, imaging with [^99m^Tc]Tc-mebrofenin can be affected by high blood bilirubin levels as both substrates compete for the same uptake transporter on the hepatocytes [94]. Both imaging methods are capable of estimating both the global and regional liver function with the use of SPECT. No head-to-head comparative studies of the two tracers in the same population were identified.

Hepatobiliary scintigraphy has shown a promising value in the preoperative assessment of patients undergoing liver resection, and it has been utilized by several nuclear medicine departments worldwide over the last decade. Still, there are no widely accepted international guidelines that recommend the use of nuclear medicine imaging to determine the resectability of patients prior to undergoing a procedure with the purpose of removing diseased liver tissue. CT volumetry, however, is well established for the preoperative estimation of FLR volume prior to liver surgery and is the present gold standard to determine resectability in patients undergoing liver resection. Preoperative CT volumetry cut-off values for a safe liver resection have been established on the basis of several well-designed studies [95–101]. It is generally accepted that approximately 75–80% of the total liver volume can be removed safely without postoperative complications, leaving a FLR of approximately 20–25% if the liver morphology and liver function are normal [95–101]. If the normal liver has been subjected to chemotherapy recently, a FLR volume of 30% is necessary, and in patients with cirrhosis or hepatitis, the FLR volume needs to be at least 40%, depending on the total liver function measured by a variety of tests and scores [99–101].

CT volumetry is, however, an indirect measure of liver function due to the heterogeneously distributed liver tissue, especially in patients with parenchymal liver disease. Therefore, using CT volumetry as a preoperative measure of the FLR requires knowledge of the quality of the liver parenchyma [11] as reflected in the different cut-off values for the various liver conditions. In addition, in using CT volumetry as the preoperative assessment of the FLR, some patients are at risk of being excluded from surgery with curative intent due to a small FLR volume, even if their actual FLR function is sufficient. Other patients with sufficient FLR volume are at risk of developing postoperative liver failure due to insufficient FLR function. To avoid these problems, a more direct evaluation of FLR function is needed, and nuclear imaging seems to be the most promising approach. Furthermore, with the use of nuclear imaging as a preoperative measure of the FLR function, one cut-off level for a safe liver resection might suffice for all patients regardless of the underlying liver function [10, 11]. Therefore, it would be advantageous for clinicians to estimate the function of the FLR and thus the postprocedural clinical course of the patients while taking into account the underlying liver disease.

For the nuclear imaging techniques to be included in the diagnostic assessment of patients undergoing liver-directed treatments, clinicians need evidence-based cut-off values for the nuclear imaging liver functional assessment based on a validated and simple liver function uptake calculation. Most cut-off values shown in this systematic review were post hoc, data-driven values, not prospective assessments of a fixed cut-off value. It was evident that nuclear imaging techniques play a promising role in the preprocedural work-up of patients undergoing liver-directed treatments, especially for the prediction of postoperative liver failure. However, the liver failure and mortality predictions are influenced by the different and inconsistent liver failure and mortality definitions. The abundant heterogeneity in nuclear imaging techniques, acquisition methods, and outcome definitions complicate the ability to establish evidence-based guidelines for the preprocedural work-up of patients undergoing liver-directed treatment. External validation and comparison of the results across the different studies require procedural standardization, both of technical performance and outcome. Rassam et al. have described practical guidelines on how to use [^99m^Tc]Tc-mebrofenin hepatobiliary scintigraphy [102]. Most papers investigated both major and minor surgery in the same study and established a joint cut-off for a safe liver resection irrespective of the surgery type. However, the extent of liver surgery may affect the FLR cut-off needed for a safe surgery and a favorable postoperative outcome. Therefore, future studies should distinguish between major and minor surgery in their prediction models and cut-off establishments. It remains unclear if the same cut-off can be used across different type of interventions.

Due to the interesting data generated with the tracers [^99m^Tc]Tc-GSA and [^99m^Tc]Tc-mebrofenin, prospective trials with predefined and clinically relevant definitions of mortality and liver failure are warranted. However, [^99m^Tc]Tc-GSA is not yet commercially available outside of Japan and there are currently no head-to-head studies comparing this tracer with [^99m^Tc]Tc-mebrofenin. The two tracers should be directly compared in prospective, multicenter studies in patients undergoing major liver surgery using predefined definitions of liver failure and mortality; the predictive findings should be compared to the gold standard of CT volumetry. This would hopefully lead to an extraction of clinically relevant cut-off levels of the nuclear imaging techniques for a safe hepatectomy.

This up-to-date systematic review covered four decades of research published in two major medical databases, employed extensive and detailed research strings, and had two investigators throughout the review and data extraction process. The authors consider this systematic review to encompass the first synthesized evidence on this clinically relevant topic.

Some groups published several, similarly appearing papers; some groups published papers with increasing number of subjects over time. We did not contact individual authors to investigate any overlapping data among papers from the same institution. This may cause a potential bias of duplicate or overlapping data if authors did not comply with established guidelines on the ethics of publishing.

## Conclusion

In conclusion, more than 80 trials have been published on preoperative nuclear medicine methods, predominantly [^99m^Tc]Tc-GSA and [^99m^Tc]Tc-mebrofenin, to predict clinical outcomes after the liver-directed treatment of non-systemic liver diseases. Even though we identified evidence of benefit for preoperative nuclear medicine assessment across various liver diseases, the data were very heterogeneous concerning the methodology for liver function uptake calculations and dissimilar outcome definitions of LF and mortality. The general impression was that this area of research is short of confirmatory and consistent evidence to determine the patient-relevant benefit of the preoperative assessment of the postoperative FLR with nuclear medicine tests. We encourage the nuclear medicine society in collaboration with hepatobiliary surgeons to support prospective, multi-national clinical efficacy trials documenting that adding a preoperative nuclear medicine test benefits patients in comparison to the standard of care for preoperative investigations using CT volumetry.

## Supplementary information

**Additional file 1:.** Supplementary files

## Data Availability

All data generated or analyzed during this study are included in this published article [and its supplementary information files].

## Abbreviations

GSA: (Human) galactosyl serum albumin
LF: Liver failure
FLR: Future liver remnant
PICOS criteria: Patient, intervention, comparison, outcome, study design criteria
PRISMA guidelines: Preferred Reporting Items for Systematic Reviews and Meta-Analyses guidelines
OR: Odds ratio
HR: Hazard ratio
