# Supplementary material for: Nuclear imaging methods for the prediction of postoperative morbidity and mortality in patients undergoing localized, liver-directed treatments: a systematic review
Source: EJNMMI Res. 2020 Sep 4;10:101. doi: 10.1186/s13550-020-00687-1 (PMC7474046; doi:10.1186/s13550-020-00687-1)
Supplement: Supplementary file 1 — Additional file 1:. Supplementary files [file 13550_2020_687_MOESM1_ESM.docx]

**Supplementary files**

***Supplementary file 1.***

| **PICOS-subjects** | **Criteria for the systematic review** |
| --- | --- |
| Patients | Patients who will undergo any local treatment of focal or multifocal liver/hepatic parenchyma with an intent to destroy or eliminate the diseased liver tissue, irrespective of the underlying cause of disease. |
| Liver intervention methods | May include, but not limited to surgery (e.g., lobectomy, segmentectomy, subsegmentomy, right or left hepatectomy, partial liver resection), radiotherapy (external or internal [with radioisotope] radiotherapy), cryotherapy, percutaneous ethanol injection, percutaneous microwave coagulation therapy, radiofrequency ablation, or transcatheter arterial chemoembolization. |
| Imaging intervention methods | Preoperative assessment of liver function by a nuclear medicine technique (requires the use of a radio-isotope labeled compound AND imaging of the liver function) with a purpose to predict postintervention clinical outcome or estimate the postintervention liver function (see above). It may include but is not limited to one of the following nuclear imaging methods:   - [^99m^Tc]Tc-Hepatic Iminodiacetic Acid ([^99m^Tc]Tc-HIDA) scintigraphy (either planar or with SPECT) - [^99m^Tc]Tc-Mebrofenin scintigraphy (either planar or with SPECT) - [^99m^Tc]Tc-Galactosyl Human Serum Albumin ([^99m^Tc]Tc-GSA) scintigraphy (either planar or with SPECT) - 2-[^18^F]fluoro-2-deoxygalactose ([^18^F]-FDGal) PET-scan |
| Comparison | None required for the PICO.  If available in the eligible papers, the nuclear medicine methods will be compared to other reported measures of liver function, e.g., CT-volumetry. |
| Outcome | At least one of the following indicators of clinical outcome or liver function post-intervention:   - Mortality (by example, but not limited to overall survival [90-day mortality rate preferred, but not mandatory], recurrence free survival, etc.) - Presence of liver failure (by example, but not limited to 50-50 method of evaluating liver function postoperatively [e.g., Balzan et al, Ann Surg. 2005;242:824-8]) - Presence of and time to clinical or biochemical liver failure - The presence (and/or graded evaluation) of postintervention complications and morbidities (by example, but not limited to ascites, hepatic encephalopathy, hepatorenal syndrome and hyperbilirubinemia) - Biochemical liver function parameters (by example, but not limited to serum cholinesterase activity, serum total bilirubin, prothrombin time, serum albumin, hepaplastin test, plasma ammonia levels, aspartate aminotransferase, alanine aminotransferase, alkaline phosphatase, gamma-glutamyltransferase, and albumin levels) - Other liver function tests postintervention (by example, but not limited to ICG-clearance, Amino Acid Clearance Test and GEC Test) - Nuclear medicine imaging liver function assessment - Duration of hospital stay - Quality of life - Performance score   The postintervention outcome has to be correlated to the preintervention nuclear medicine imaging technique, such that the imaging examination either selects patients for the intervention (with a predetermined cut-off level) or the outcome is presented as a function of the result of the preintervention nuclear medicine imaging technique. |
| Study design | Any study type with a minimum of 5 patients per study |

**Supplementary file 2.**

# Literature search strategy

The MEDLINE (Ovid) and Web of Science bibliographic databases:

| Database | Interface | Number of hits on May 27, 2020 |
| --- | --- | --- |
| MEDLINE | Ovid | 653 |
| Web of Science |  | 691 |

### MEDLINE (Ovid) June 20, 2017

| ID | Search terms |
| --- | --- |
| 1 | exp preoperative period/ |
| 2 | preoperative*.mp. |
| 3 | pre-operative*.mp. |
| 4 | Predictive model*.mp |
| 5 | 1 or 2 or 3 or 4 |
| 6 | exp Biliary Tract Neoplasms/ |
| 7 | (biliary adj3 cancer*).mp. |
| 8 | (biliary adj3 neoplasm*).mp. |
| 9 | (biliary adj3 adenom*).mp. |
| 10 | (biliary adj3 metastas*).mp. |
| 11 | (biliary adj3 cystadeno*).mp. |
| 12 | (biliary adj3 hemangiom*).mp. |
| 13 | (biliary adj3 carcinom*).mp. |
| 14 | (biliary adj3 tumo?r*).mp. |
| 15 | (bile duct adj3 cancer*).mp. |
| 16 | (bile duct adj3 neoplasm*).mp. |
| 17 | (bile duct adj3 adenom*).mp. |
| 18 | (bile duct adj3 metastas*).mp. |
| 19 | (bile duct adj3 cystadeno*).mp. |
| 20 | (bile duct adj3 hemangiom*).mp. |
| 21 | (bile duct adj3 carcinom*).mp. |
| 22 | (bile duct adj3 tumo?r*).mp. |
| 23 | (gallbladder adj3 cancer*).mp. |
| 24 | (gallbladder adj3 neoplasm*).mp. |
| 25 | (gallbladder adj3 adenom*).mp. |
| 26 | (gallbladder adj3 metastas*).mp. |
| 27 | (gallbladder adj3 cystadeno*).mp. |
| 28 | (gallbladder adj3 hemangiom*).mp. |
| 29 | (gallbladder adj3 carcinom*).mp. |
| 30 | (gallbladder adj3 tumo?r*).mp. |
| 31 | (hepatic adj3 cancer*).mp. |
| 32 | (hepatic adj3 neoplasm*).mp. |
| 33 | (hepatic adj3 adenom*).mp. |
| 34 | (hepatic adj3 metastas*).mp. |
| 35 | (hepatic adj3 cystadeno*).mp. |
| 36 | (hepatic adj3 hemangiom*).mp. |
| 37 | (hepatic adj3 carcinom*).mp. |
| 38 | (hepatic adj3 tumo?r*).mp. |
| 39 | exp Liver Diseases/ |
| 40 | Remnant liver.mp |
| 41 | FRL.mp |
| 42 | exp Liver Function Tests/ |
| 43 | Liver function.mp |
| 44 | or/6-43 |
| 45 | exp General Surgery/ |
| 46 | surger*.mp. |
| 47 | lobectom*.mp. |
| 48 | segmentom*.mp. |
| 49 | exp Hepatectomy/ |
| 50 | (liver adj3 resection*).mp. |
| 51 | (hepatic adj3 resection*).mp. |
| 52 | exp Radiotherapy/ |
| 53 | Radiotherap*.mp. |
| 54 | Radiation therap*.mp. |
| 55 | irradiation*.mp. |
| 56 | Brachytherapy.mp. |
| 57 | Chemoradiotherap*.mp. |
| 58 | Radiochemotherap*.mp. |
| 59 | Immunoradiotherap*.mp. |
| 60 | Radioimmunotherap*.mp. |
| 61 | Beam therap*.mp. |
| 62 | Proton therap*.mp. |
| 63 | radiofrequency ablation.mp. |
| 64 | exp Cryotherapy/ |
| 65 | cryotherap*.mp. |
| 66 | cold therap*.mp. |
| 67 | induced hypothermia.mp. |
| 68 | percutaneous ethanol injection*.mp. |
| 69 | percutaneous microwave coagulation therap*.mp. |
| 70 | transcatheter* chemoemboli*.mp. |
| 71 | or/45-70 |
| 72 | exp Fluorodeoxyglucose F18/ |
| 73 | 18f fluorodeoxyglucose.mp. |
| 74 | 18f-fdg.mp. |
| 75 | 18fdg.mp. |
| 76 | 2 fluoro 2 deoxy d glucose.mp. |
| 77 | 2 fluoro 2 deoxyglucose.mp. |
| 78 | fludeoxyglucose f 18.mp. |
| 79 | fludeoxyglucose f18.mp. |
| 80 | fluorodeoxyglucose f 18.mp. |
| 81 | fluorodeoxyglucose f18.mp. |
| 82 | fluorine 18 fluorodeoxyglucose.mp. |
| 83 | exp radiopharmaceuticals/ |
| 84 | exp Positron-Emission Tomography/ |
| 85 | Positron Emission Tomography.mp. |
| 86 | pet scan*.mp. |
| 87 | exp Radionuclide Imaging/ |
| 88 | radionuclide imaging.mp. |
| 89 | scintigraph*.mp. |
| 90 | scintiphotograph*.mp. |
| 91 | (gamma adj3 imaging).mp. |
| 92 | 99m* tc*.mp. |
| 93 | tc* 99m*.mp. |
| 94 | 99mtc*.mp. |
| 95 | SPECT*.mp. |
| 96 | or/72-95 |
| 97 | 5 and 44 and 71 and 96 |

### Web of Science Core Collection May 27, 2020

| ID | Search terms |
| --- | --- |
| 1 | TS=((liver OR hepatic OR gallbladder OR biliary OR (bile duct)) AND (cancer* OR neoplasm* OR adenocarcinoma* OR carcinoma* OR neoplasia OR tumor OR tumors OR tumour OR tumours))  DocType=All document types; Language=All languages; |
| 2 | TS=(preoperative OR (pre operative) OR (predictive model*))  DocType=All document types; Language=All languages; |
| 3 | TS=(surger* OR lobectom* OR segmentom* OR hepatectom* OR (liver resection*) OR (hepatic resection*) OR radiotherap* OR (radiation therap*) OR irradiation* OR Brachytherapy OR Chemoradiotherap* OR Radiochemotherap* OR Immunoradiotherap* OR radioimmunotherap* OR (Beam therap*) OR (proton therap*) OR (radiofrequency ablation) OR cryotherap* OR (cold therap*) OR (induced hypothermia) OR (percutaneous ethanol injection*) OR (percutaneous microwave coagulation therap*) OR (transcatheter* chemoemboli*))  DocType=All document types; Language=All languages; |
| 4 | TS=(Fluorodeoxyglucose OR 18fdg* OR (18f fdg*) OR fludeoxyglucose OR radiopharmaceutical* OR (pet scan*) OR (positron emission tomograph*) OR (radionuclide imaging) OR scintiscan* OR SPECT* OR scintiphotograph* OR (gamma imaging) OR 99mtc* OR (tc 99m*))  DocType=All document types; Language=All languages; |
| 5 | 1 AND 2 AND 3 AND 4 |
| 6 | TS=(remnant liver OR FRL OR liver function) |
| 7 | 6 OR 1 |
| 8 | 7 AND 4 AND 3 AND 2 |

### 
